# The physical and mental health of a large military cohort: baseline functional health status of the Millennium Cohort

**DOI:** 10.1186/1471-2458-7-340

**Published:** 2007-11-26

**Authors:** Tyler C Smith, Mark Zamorski, Besa Smith, James R Riddle, Cynthia A LeardMann, Timothy S Wells, Charles C Engel, Charles W Hoge, Joyce Adkins, Dan Blaze, the Millennium Cohort Study Team

**Affiliations:** 1 Department of Defense Center for Deployment Health Research, Naval Health Research Center, San Diego, CA, USA.; 2 Deployment Health Section, Directorate of Medical Policy, Canadian Forces Health Services Group Headquarters and Department of Family Medicine, University of Ottawa, Ottawa, Ontario, Canada.; 3 Air Force Research Laboratory, Wright-Patterson Air Force Base, OH, USA.; 4 Deployment Health Clinical Center, Walter Reed Army Medical Center, Silver Spring, MD, USA.; 5 Department of Psychiatry and Behavioral Sciences, Walter Reed Army Institute of Research, Silver Spring, MD, USA.; 6 Office of the Assistant Secretary of Defense for Health Affairs, Force Health Protection, the Pentagon, Washington, DC, USA.; 7 Duke University Medical Center, Durham, NC, USA.

## Abstract

**Background:**

The US military is currently involved in large, lengthy, and complex combat operations around the world. Effective military operations require optimal health of deployed service members, and both mental and physical health can be affected by military operations.

**Methods:**

Baseline data were collected from 77,047 US service members during 2001–2003 as part of a large, longitudinal, population-based military health study (the Millennium Cohort Study). The authors calculated unadjusted, adjusted, and weighted means for the Medical Outcomes Study Short Form 36-item Survey for Veterans physical (PCS) and mental component summary (MCS) scores over a variety of demographic and military characteristics at baseline.

**Results:**

The unadjusted mean PCS and MCS scores for this study were 53.4 (95% confidence interval: 53.3–53.4) and 52.8 (95% confidence interval: 52.7–52.9). Average PCS and MCS scores were slightly more favorable in this military sample compared to those of the US general population of the same age and sex. Factors independently associated with more favorable health status included male gender, being married, higher educational attainment, higher military rank, and Air Force service. Combat specialists had similar health status compared to other military occupations. Having been deployed to Southwest Asia, Bosnia, or Kosovo between 1998 and 2000 was not associated with diminished health status.

**Conclusion:**

The baseline health status of this large population-based military cohort is better than that of the US general population of the same age and sex distribution over the same time period, especially in older age groups. Deployment experiences during the period of 1998–2001 were not associated with decreased health status. These data will serve as a useful reference for other military health studies and for future longitudinal analyses.

## Background

Military operations demand optimal physical and mental health. Despite technological advances, operations continue to demand a high level of fitness and physical functioning. While serious physical health problems are uncommon in young and middle-aged adults who make up the bulk of the American military, mental health problems are prevalent in this age group in the general population. Common mental health problems such as mood and anxiety disorders can interfere with the substantial energy, concentration, motivation, and judgment required for success of the military mission. Technological advances and the complex nature of recent conflicts have, if anything, increased demands in these areas. To the extent that optimal physical and mental health are required to defend the interests of a nation, the health of service members is a matter of national security.

At least some military operations can result in mental and physical health deterioration. A substantial burden of mental health problems was identified in Vietnam War veterans, and Persian Gulf War veterans reported a multitude of physical and psychological symptoms and illnesses at rates two to three times higher than nondeployed veterans of the same era [1-6]. The health-related quality of life reported by these veterans has also been shown to be significantly less favorable [2,7-9].

Increased risk of mental health problems and physical symptom reporting by war veterans has been common following other major conflicts [10]. While there is a consensus that war trauma can lead to measurable adverse mental and physical health effects, emerging data suggest that not all deployments have the same propensity to cause these problems. For example, UK veterans from the Bosnian conflict had much better health than UK Gulf War veterans [7]. A recent survey of UK veterans of the current Iraq conflict showed surprisingly little difference in their physical or mental health status relative to nondeployed controls [11,12]. However, a limitation in some of the research following the 1991 Gulf War and more recent conflicts was that comparison populations were unsuitable due to differences in health and composition of deployed and nondeployed personnel [13,14]. While researchers were diligent in documenting these limitations [15], there is an obvious and growing need for baseline data from which to answer the health concerns of veterans.

The Millennium Cohort Study was launched in October 2000 [16], in response to the US Department of Defense recommendation for a coordinated effort to study the potential health effects of deployment-related exposures [17], and the Institute of Medicine recommendation for a systematic, longitudinal, population-based assessment of service members' health [18]. The Cohort began enrollment for the 21-year longitudinal study in July 2001 and completed enrolling Panel 1 participants in June 2003.

This report investigates the baseline mental and physical health status of this large military cohort, as measured by the Medical Outcomes Study Short Form 36-item Survey for Veterans [19]. Health status as measured by the Medical Outcomes Study Short Form 36-item Survey for Veterans and its parent form (SF-36) have been associated with increased health care utilization [20-22], posttraumatic stress disorder [23], disability [22], behavioral risk factors [22], and mortality [20,22] in US military veterans. Aside from their value as a baseline for future analyses on this cohort, these data are crucial to understanding the health of US military personnel in several ways: These population-level data can be used to compare the health of the military to other populations, such as the US general population. Baseline cross-sectional data presented in this report will be useful as a reference for exploring the health of particular subgroups within the military, such as those in a particular military occupation or those who deployed to a particular geographical area. Identification of risk groups within the Millennium Cohort will also help target those service groups for interventions; the identification of these risk groups can also be used to generate hypotheses as to how military service influences health status.

## Methods

### Study population

The Millennium Cohort Study Panel 1 consists of 77,047 consenting military service members who were enrolled using a modified Dillman approach [24] and offered both Web and US postal-based submission options (36% response rate of those invited to participate) [16]. The invited Cohort was sampled from electronic personnel records representing approximately 11.3 percent of the 2.2 million men and women in service as of October 1, 2000. Enrollment began in July 2001 and ended in June 2003. US military personnel serving in the Army, Navy, Coast Guard, Air Force, and Marine Corps were selected and oversampled for those with recent deployment experience to Southwest Asia, Bosnia, or Kosovo between 1998 and 2000, Reserve and National Guard members, and female service members to ensure adequate power for statistical inferences over the 21-year follow-up period. The Millennium Cohort baseline enrollment, designed to invite sufficient numbers of women and recent deployers has been shown to be representative in composition of the US military [16]. Investigation of potential biases in the Millennium Cohort Study have found no differences in healthcare utilization, as measured by hospital encounters and outpatient care, prior to study invitation as a determinant for enrollment (data not yet published). Investigation of Millennium Cohort self-reported data have been found reliable in test re-test investigations and when comparing to electronically maintained databases and to have high internal consistency within standardized instruments [16,25-31]. This research has been conducted in compliance with all applicable federal regulations governing the protection of human subjects in research and was reviewed by the Naval Health Research Center Institutional Review Board as Protocol NHRC.2000.0007.

Demographic data for the Millennium Cohort Study participants reflect status as of October 1, 2000. Data included gender, date of birth (age in years: 17–24, 25–34, 35–44, > 44), education level (no high school diploma, high school diploma, some college, college degree), marital status (never married, married, divorced), race/ethnicity (White non-Hispanic, Black non-Hispanic, Asian/Pacific Islander, Hispanic, and other), length of service (in years: 0–3, 4–8, 9–15, ≥ 16), military rank (enlisted, warrant officer, commissioned officer), service component (Reserve/National Guard, active duty), service branch (Army, Air Force, Navy/Coast Guard, Marines), and US Department of Defense primary and duty occupations (combat specialist, other occupations) [32]. Additionally, Cohort members were identified with past deployment experience to Southwest Asia, Bosnia, or Kosovo during the period of January 1, 1998, to September 1, 2000. For this study, missing demographic data for marital status, occupation, education, and rank were supplemented with self-reported data from the survey when possible. This reduced those missing data for at least one demographic characteristic from 1.8 percent to 0.7 percent of the Cohort.

### Outcomes

The Millennium Cohort Study questionnaire consists of more than 450 questions and components regarding diagnosed medical conditions, reported symptoms, psychosocial assessment, physical status, functional status, alcohol use, tobacco use, occupation, alternative medicine use, exposures, sleep patterns, deployment experience, and basic demographic and contact data [16]. This paper focuses on self-reported health status as measured by Medical Outcomes Study Short Form 36-item Survey for Veterans [19], a modified version of the Medical Outcomes Study 36-item Short Form Health Survey (SF-36) [33]. Like the original SF-36, the Veterans SF-36 includes eight health scales which can be summarized into two summary scores, the mental component summary (MCS) and the physical component summary (PCS) [34-37]. Differences in the Veterans SF-36 include using 5-level response categories for the role emotional and role physical scales which provides more-precise estimates of role functional impairment [38,39]. A validated approach was used to create the MCS and PCS scores, that uses the same factor weights, general population means, and standard deviations as the original SF-36 scoring mechanism, which make the scores comparable to the original SF-36 version [38]. The PCS and MCS normative US scores have a mean of 50 and a standard deviation of 10, allowing comparison to other populations [34,35]. Higher MCS and PCS scores reflect more favorable health status.

### Statistical analyses

Descriptive investigation of Cohort characteristics compared with the invited participants and the 2000 US military were completed. Unadjusted aggregated Cohort means were computed for the two summary components. Multicollinearity was investigated among the variables age, sex, education, marital status, race/ethnicity, recent deployment to Southwest Asia, Bosnia, or Kosovo, length of service, military rank, branch of service, and occupational category. These variables were included in an analysis of variance (ANOVA) to calculate adjusted means for the two summary component scores with the Tukey approach to adjust for multiplicity [40,41].

Although Cohort proportions and 2000 US military proportions of Reserve and National Guard members are similar, the initial sampling design of the Millennium Cohort oversampled for female, recently deployed, and Reserve/National Guard members. To account for designed oversampling for these characteristics, weighted means for subgroups of the population were calculated based on the inverse of the sampling fraction for the three characteristics oversampled: female, recently deployed, and Reserve/National Guard member. Variance was estimated using the Taylor series expansion theory to estimate sampling errors based on the complex sampling [42]. Data management, ANOVA, weighted, and nonweighted analyses were completed using SAS^® ^software (Version 9.1, SAS Institute, Inc., Cary, North Carolina) [43].

## Results

Of the 77,047 Cohort participants, complete demographic and questionnaire data for the MCS and PCS scores were available for 75,413 (97.9%). The study population consisted of US military personnel proportionately more likely to be: 35 or older, college educated, married, White non-Hispanic, in service more than 8 years, and in the officer ranks (Table 1). Chi-square tests of association suggest statistically significant differences in composition between the 2000 US military, the invited Cohort and the Cohort members, although much of the difference is due to the sampling strategy employed [16].

**Table 1:** Characteristics of Millennium Cohort Study Members at Baseline and the US Military in October 2000

Characteristic*	Cohort^† ^N = 75,413 *n *(%)		Invited Cohort^‡ ^N = 207,683 (%)	US Military^§ ^N = 2,140,959 (%)
Sex				
Male	55,307	(73.3)	(76.3)	(84.8)
Female	20,106	(26.7)	(23.7)	(15.2)
Age, years				
17–24	14,249	(18.9)	(27.8)	(32.1)
25–34	26,479	(35.1)	(35.8)	(34.0)
35–44	24,891	(33.0)	(27.0)	(25.3)
> 44	9,794	(13.0)	(9.5)	(8.6)
Education				
No high school diploma	4,620	(6.1)	(7.5)	(8.2)
High school diploma diploma/equivalent	32,329	(42.9)	(49.1)	(53.3)
Some college	19,333	(25.6)	(25.0)	(21.0)
College degree	19,131	(25.4)	(18.5)	(17.4)
Marital status				
Never married	22,568	(29.9)	(38.3)	(41.0)
Married	47,680	(63.2)	(55.6)	(54.0)
Divorced	5,165	(6.9)	(6.1)	(5.1)
Race/ethnicity				
White non-Hispanic	52,613	(69.8)	(66.0)	(67.8)
Black non-Hispanic	10,294	(13.7)	(18.3)	(18.9)
Asian/Pacific Islander	5,983	(7.9)	(6.3)	(3.3)
Hispanic	4,838	(6.4)	(7.1)	(7.9)
Other	1,685	(2.2)	(2.3)	(2.1)
1998–2000 deployment to Bosnia, Kosovo, or Southwest Asia				
No	52,590	(69.7)	(70.0)	(90.0)
Yes	22,823	(30.3)	(30.0)	(10.0)
Length of service, years				
0–3	13,661	(18.1)	(21.9)	(31.0)
4–8	17,042	(22.6)	(26.7)	(21.0)
9–15	19,530	(25.9)	(23.8)	(21.0)
≥ 16	25,180	(33.4)	(27.5)	(27.1)
Military rank				
Enlisted	58,228	(77.2)	(83.9)	(84.6)
Warrant officer	1,345	(1.8)	(1.2)	(1.1)
Commissioned officer	15,840	(21.0)	(15.0)	(14.2)
Service component				
Reserve/National Guard	32,418	(43.0)	(47.3)	(41.5)
Active duty	42,995	(57.0)	(52.7)	(58.5)
Branch of service				
Army	35,773	(47.4)	(44.9)	(46.5)
Air Force	22,074	(29.3)	(29.6)	(24.3)
Navy and Coast Guard	13,696	(18.2)	(19.3)	(20.5)
Marines	3,870	(5.1)	(6.2)	(8.6)
Occupational category				
Combat specialists	15,083	(20.0)	(20.8)	(22.3)
Other occupations	60,330	(80.0)	(79.2)	(77.7)

* All characteristics are significantly different between the Cohort, Invited Cohort, and US Military (p < .05).

^† ^Only participants with complete demographic data, mental component summary and physical component summary scores were included in this study.

^‡ ^Includes invited members who were contacted by US Postal Service mail at least one time and had complete demographic data, with the exception of length of service.

^§ ^Based on US military service rosters of October 2000 with complete demographic data.

Table 2 reports the unadjusted means for the MCS and PCS scores stratified by gender. All scores are above the US population mean of 50, with the exception of mean MCS scores for women in a few subgroups including those who are younger, less educated, serving in the Marine Corps and who have 3 years or less of service. The weighted and adjusted subgroup means for the MCS and PCS scores are reported in Table 3. The weighted subgroup means are useful for identifying strata at increased risk for decreased mental and physical health status, while the adjusted subgroup means better reflect the independent contribution of the risk factors. The adjusted and weighted subgroup means showed virtually identical patterns of risk factors, though, as expected, the adjusted means showed less dramatic differences.

**Table 2:** Unadjusted Mental and Physical Component Summary Scores* for Millennium Cohort Study Participants (N = 75,413)

	MCS		PCS	
	
Characteristic	Males	Females	Males	Females
Full Cohort	53.4 (53.3, 53.5)	51.1 (50.9, 51.2)	53.6 (53.5, 53.6)	52.9 (52.8,53.0)

Age, years				
17–24	50.8 (50.5, 51.0)	48.9 (48.6, 49.2)	54.2 (54.1, 54.4)	53.1 (52.8, 53.3)
25–34	53.2 (53.0, 53.3)	51.0 (50.7, 51.2)	54.2 (54.1, 54.3)	53.5 (53.3, 53.7)
35–44	54.2 (54.1, 54.3)	52.3 (52.0, 52.6)	53.0 (52.9, 53.1)	52.4 (52.2, 52.7)
≥ 44	55.2 (55.0, 55.4)	53.6 (53.2, 54.0)	52.7 (52.5, 52.8)	52.1 (51.7, 52.4)
Education				
No high school diploma	51.9 (51.5, 52.3)	49.9 (49.4, 50.5)	52.9 (52.6, 53.2)	52.6 (52.2, 53.1)
High school diploma diploma/equivalent	52.3 (52.2, 52.5)	49.7 (49.4, 49.9)	52.9 (52.8, 53.0)	52.0 (51.8, 52.2)
Some college	54.0 (53.9, 54.2)	51.8 (51.5, 52.0)	53.4 (53.3, 53.5)	53.2 (53.0, 53.4)
College degree	55.0 (54.9, 55.2)	53.0 (52.8, 53.3)	55.1 (55.0, 55.2)	54.3 (54.1, 54.5)
Marital status				
Never married	51.6 (51.5, 51.8)	50.3 (50.1, 50.6)	54.4 (54.3, 54.6)	53.2 (53.1, 53.4)
Married	54.1 (54.0, 54.2)	51.7 (51.5, 51.9)	53.3 (53.2, 53.3)	52.7 (52.6, 52.9)
Divorced	53.4 (53.1, 53.8)	51.2 (50.7, 51.6)	53.2 (52.9, 53.5)	52.5 (52.1, 52.8)
Race/ethnicity				
White non-Hispanic	53.2 (53.2, 53.3)	50.8 (50.6, 51.0)	53.6 (53.5, 53.7)	53.2 (53.1, 53.4)
Black non-Hispanic	54.2 (54.0, 54.4)	51.8 (51.5, 52.2)	52.8 (52.6, 53.0)	51.8 (51.6, 52.1)
Asian/Pacific Islander	54.3 (54.1, 54.6)	52.3 (51.8, 52.8)	54.3 (54.1, 54.5)	53.8 (53.4, 54.2)
Hispanic	53.1 (52.8, 53.4)	50.3 (49.7, 50.9)	53.6 (53.3, 53.8)	52.9 (52.5, 53.4)
Other	53.0 (52.4, 53.5)	50.2 (49.3, 51.2)	53.0 (52.6, 53.5)	52.0 (51.3, 52.8)
1998–2000 deployment to Bosnia, Kosovo, or Southwest Asia				
No	53.4 (53.3, 53.5)	51.1 (50.9, 51.2)	53.6 (53.5, 53.7)	52.9 (52.8, 53.1)
Yes	53.5 (53.4, 53.7)	51.3 (50.9, 51.7)	53.5 (53.4, 53.6)	52.8 (52.5, 53.1)
Length of service, years				
0–3	51.2 (50.9, 51.4)	49.4 (49.1, 49.7)	54.3 (54.2, 54.5)	53.1 (52.9, 53.3)
4–8	52.6 (52.4, 52.8)	50.5 (50.2, 50.8)	54.3 (54.1, 54.4)	53.4 (53.2, 53.6)
9–15	53.9 (53.7, 54.0)	52.1 (51.8, 52.4)	54.0 (53.9, 54.1)	53.4 (53.1, 53.6)
≥ 16	54.5 (54.4, 54.6)	52.6 (52.3, 52.9)	52.5 (52.4, 52.6)	51.8 (51.6, 52.1)
Military rank				
Enlisted	52.9 (52.8, 53.0)	50.4 (50.3, 50.6)	53.1 (53.0, 53.1)	52.5 (52.3, 52.6)
Warrant officer	55.4 (55.0, 55.9)	54.0 (52.6, 55.4)	52.9 (52.5, 53.3)	51.6 (50.3, 52.9)
Commissioned officer	55.3 (55.1, 55.4)	53.4 (53.1, 53.6)	55.5 (55.4, 55.6)	54.6 (54.4, 54.8)
Service component				
Reserve/National Guard	53.8 (53.7, 53.9)	51.8 (51.5, 52.0)	54.1 (54.0, 54.2)	53.7 (53.5, 53.8)
Active duty	53.1 (53.0, 53.2)	50.5 (50.3, 50.7)	53.2 (53.1, 53.3)	52.2 (52.0, 52.4)
Branch of service				
Army	53.0 (52.9, 53.1)	50.7 (50.5, 51.0)	53.1 (53.0, 53.2)	52.1 (52.0, 52.3)
Air Force	54.5 (54.3, 54.6)	52.2 (52.0, 52.5)	54.2 (54.0, 54.3)	54.1 (53.9, 54.3)
Navy and Coast Guard	53.1 (52.9, 53.3)	50.4 (50.0, 50.8)	53.8 (53.7, 54.0)	53.2 (52.9, 53.4)
Marines	52.7 (52.4, 53.1)	49.6 (48.6, 50.6)	53.8 (53.6, 54.1)	52.6 (51.8, 53.4)
Occupational category				
Combat specialists	53.8 (53.7, 53.9)	51.0 (50.4, 51.6)	54.1 (54.0, 54.2)	53.3 (52.9, 53.8)
Other occupations	53.3 (53.2, 53.4)	51.1 (50.9, 51.3)	53.4 (53.3, 53.5)	52.9 (52.8, 53.0)

* Medical Outcomes Study 36-Item Short Form Health Survey for Veterans. 1998 general US population means used to calculate mental and physical component summary scores (MCS, PCS).

**Table 3:** Adjusted and Weighted Mental and Physical Component Summary Score Means for Millennium Cohort Study Participants (N = 75,413)

	MCS		PCS	
	
Characteristic	Adjusted Means^†^	Weighted Means^‡^	Adjusted Means	Weighted Means
Sex				
Male	54.2^a^	53.3^a^	54.5^a^	53.5^a^
Female	52.2^b^	51.0^b^	53.4^b^	52.8^b^
Age, years				
17–24	52.1^a^	50.2^a^	55.2^a^	54.0^a^
25–34	53.0^b^	52.7^b^	54.7^b^	54.1^a^
35–44	53.4^c^	53.9^c^	53.4^c^	52.8^b^
≥ 44	54.1^d^	55.1^d^	52.4^d^	52.6^c^
Education				
No high school diploma	52.6^a^	51.6^a^	53.4^a^	52.8^a^
High school diploma diploma/equivalent	53.0^a^	51.8^a^	53.5^a^	52.7^a^
Some college	53.5^b^	53.5^b^	54.1^b^	53.3^b^
College degree	53.6^b^	54.7^c^	54.8^c^	54.9^c^
Marital status				
Never married	53.1^a^	51.1^a^	54.3^a^	54.3^a^
Married	53.7^b^	53.8^b^	53.8^b^	53.1^b^
Divorced	52.7^a^	52.7^c^	53.8^b^	52.9^b^
Race/ethnicity				
White non-Hispanic	52.6^a^	52.8^a^	53.8^a, c^	53.5^a^
Black non-Hispanic	54.2^b^	53.5^b^	53.7^a^	52.5^b^
Asian/Pacific Islander	53.3^c^	54.1^c^	54.5^b^	54.1^c^
Hispanic	53.1^c^	52.6^a^	54.1^b, c^	53.4^a^
Other	52.7^a, c^	52.3^a^	53.5^a^	52.7^b^
1998–2000 deployment to Bosnia, Kosovo, or Southwest Asia				
No	53.1^a^	52.9^a^	54.0^a^	53.4^a^
Yes	53.3^b^	53.4^b^	53.9^a^	53.6^b^
Length of service, years				
0–3	52.6^a^	50.6^a^	54.0^a^	54.1^a^
4–8	53.0^b^	52.2^b^	54.1^a^	54.1^a^
9–15	53.5^c^	53.5^c^	54.2^a^	53.9^b^
≥ 16	53.6^c^	54.3^d^	53.4^b^	52.4^c^
Military rank				
Enlisted	52.1^a^	52.3^a^	53.1^a^	52.9^a^
Warrant officer	53.8^b^	55.2^b^	53.8^b^	52.6^a^
Commissioned officer	53.7^b^	54.9^b^	54.9^c^	55.3^b^
Service component				
Reserve/National Guard	53.5^a^	53.5^a^	55.0^a^	54.0^a^
Active duty	52.8^b^	52.6^b^	52.9^b^	53.0^b^
Branch of service				
Army	52.7^a^	52.7^a^	52.9^a^	52.9^a^
Air Force	53.8^b^	54.0^b^	54.5^b^	54.2^b^
Navy and Coast Guard	52.9^a, c^	52.6^a^	54.4^b^	53.8^c^
Marines	53.3^c^	52.4^a^	54.0^c^	53.5^d^
Occupational category				
Combat specialists	53.3^a^	53.6^a^	54.0^a^	53.9^a^
Other occupations	53.1^b^	52.8^b^	53.9^a^	53.3^b^

* Medical Outcomes Study 36-Item Short Form Health Survey for Veterans. 1998 general US population means used to calculate mental and physical component summary scores (MCS, PCS).

^† ^Means are adjusted for all variables in the table.

^‡ ^Weighting based on the inverse of the sampling scheme.

^a, b, c ^Letters that are different indicate statistically significant differences (p < 0.05) of adjusted and weighted means. Same letters indicate no statistically significant differences in means. Tukey's method was used to adjust for multiple comparisons.

Females had significantly less favorable mental and physical health status. Older participants and those with longer lengths of service had more favorable mental health but less favorable physical health. Participants with lower levels of educational attainment had less favorable mental and physical health. Overall, married participants, officers, Reserve/National Guard members, and Air Force members had significantly higher mental and physical summary scores. With the exception of adjusted PCS scores, combat specialists had slightly more favorable MCS and PCS scores. Although the difference was small, participants who had deployed to Southwest Asia, Kosovo, and Bosnia between 1998 and 2001 had slightly more favorable weighted MCS and PCS scores; this difference persisted in the adjusted MCS but not PCS scores.

To better understand gender-specific mental and physical health in the context of professional versus Reserve/National Guard or "citizen-soldiers," Table 4 presents female-only data stratified by active-duty or Reserve/National Guard status. Reserve/National Guard women had higher overall MCS and PCS scores when compared with active-duty personnel. Increasing age suggested more favorable MCS scores and less favorable PCS scores in both active-duty and Reserve/National Guard women. Increased education suggested more favorable mental health among active-duty women and more favorable physical health among Reserve/National Guard women.

**Table 4:** Adjusted Mental and Physical Component Summary Score* Means Stratified by Active Duty and Reserve/National Guard Military Women^†^

	Active Duty (n = 10,469)		Reserve/National Guard (n = 9,637)	
Characteristic	MCS	PCS	MCS	PCS

Age, years				
17–24	50.0^a^	53.5^a^	51.8^a^	55.0^a^
25–34	50.9^b^	53.2^a^	52.6^a^	54.3^b^
35–44	51.1^a, b^	51.7^b^	53.7^b^	53.2^c^
≥ 44	51.7^a, b^	49.9^c^	54.7^c^	52.1^d^
Education				
No high school diploma	48.2^a^	51.3^a, b^	52.8^a^	53.1^a^
High school diploma diploma/equivalent	51.2^b^	51.6^b^	52.9^a^	53.3^a^
Some college	52.2^c^	52.5^a^	53.6^a^	53.8^b^
College degree	52.1^b, c^	53.0^a^	53.5^a^	54.4^b^
Marital status				
Never married	51.1^a^	52.5^a^	53.9^a^	53.8^a^
Married	51.6^a^	51.9^b^	53.3^b^	53.6^a^
Divorced	50.1^b^	51.8^a, b^	52.5^c^	53.6^a^
Race/ethnicity				
White non-Hispanic	50.4^a^	51.9^a^	52.6^a^	53.9^a^
Black non-Hispanic	52.2^b^	51.6^a^	54.1^b^	53.5^a, b^
Asian/Pacific Islander	51.2^a, b^	52.9^b^	53.6^a, b^	54.6^a^
Hispanic	50.2^a^	52.3^a, b^	53.5^a, b^	53.9^a, b^
Other	50.6^a, b^	51.7^a, b^	52.3^a^	52.4^b^
1998–2000 deployment to Bosnia, Kosovo, or Southwest Asia				
No	50.6^a^	52.0^a^	53.4^a^	53.9^a^
Yes	51.3^b^	52.2^a^	53.0^a^	53.4^a^
Length of service, years				
0–3	50.0^a^	51.9^a^	53.3^a^	53.8^a^
4–8	50.7^a, b^	52.3^a, b^	52.8^a^	53.9^a^
9–15	51.4^b^	52.8^b^	53.4^a^	53.5^a^
≥ 16	51.7^b^	51.3^a^	53.3^a^	53.4^a^
Military rank				
Enlisted	49.5^a^	51.2^a^	51.8^a^	53.2^a^
Warrant officer	51.9^a, b^	51.8^a, b^	54.2^a, b^	53.1^a, b^
Commissioned officer	51.4^b^	53.3^b^	53.6^b^	54.6^b^
Branch of service				
Army	50.9^a^	50.6^a^	52.1^a^	52.7^a^
Air Force	52.0^b^	53.0^b^	53.6^b^	54.4^b^
Navy and Coast Guard	50.2^a^	52.5^b^	52.9^b^	54.0^b^
Marines	50.6^a, b^	52.1^b^	54.2^a, b^	53.5^a, b^
Occupational category				
Combat specialists	51.1^a^	52.0^a^	53.2^a^	53.5^a^
Other occupations	50.8^a^	52.2^a^	53.2^a^	53.8^a^

* Medical Outcomes Study 36-Item Short Form Health Survey for Veterans. 1998 general US population means used to calculate mental and physical component summary scores (MCS, PCS).

^† ^Only women with MCS and PCS scores and complete demographic data are reported in this table.

^a, b, c ^Letters that are different indicate statistically significant differences (p < 0.05) of adjusted and weighted means. Same letters indicate no statistically significant differences in means. Tukey's method was used to adjust for multiple comparisons.

Reserve/National Guard men had slightly more favorable mental and physical health than active-duty men (Table 5). Trends with age seen previously in women were also found in men. More favorable mental health and less favorable physical health for both active-duty and Reserve/National Guard men were found with increasing age. Higher education and being an officer were associated with more favorable mental and physical health for both active duty and Reserve/National Guard men. Combat specialty occupations were associated with more favorable mental health and physical health for Reserve/National Guard men, but only more favorable mental health for active-duty men.

**Table 5:** Adjusted Mental and Physical Component Summary Score* Means Stratified by Active Duty and Reserve/National Guard Military Men^†^

	Active Duty (n = 32,526)		Reserve/National Guard (n = 22,781)	
Characteristic	MCS	PCS	MCS	PCS

Age, years				
17–24	52.4^a^	54.6^a^	54.1^a^	56.6^a^
25–34	53.4^b^	54.3^a^	54.1^a^	55.8^b^
35–44	53.8^b^	53.2^b^	54.4^a^	54.7^c^
≥ 44	54.5^c^	52.1^c^	55.2^b^	53.6^d^
Education				
No high school diploma	52.1^a^	52.7^a^	53.9^a^	54.6^a^
High school diploma diploma/equivalent	53.6^b^	53.4^b^	54.3^a^	54.8^a^
Some college	54.3^c^	53.7^b^	54.7^b^	55.3^b^
College degree	54.1^b, c^	54.4^c^	54.9^b^	56.1^c^
Marital status				
Not married	53.2^a^	54.0^a^	54.1^a^	55.5^a^
Married	54.2^b^	53.5^b^	54.9^b^	54.8^b^
Divorced	53.2^a^	53.1^b^	54.3^a^	55.2^a, b^
Race/ethnicity				
White non-Hispanic	53.0^a^	53.3^a^	54.1^a^	55.3^a^
Black non-Hispanic	54.7^b^	53.6^a, b^	55.0^b^	54.9^a^
Asian/Pacific Islander	53.4^a^	53.9^b^	54.2^a, b^	55.6^a^
Hispanic	53.5^a^	53.9^b^	54.7^a, b^	55.0^a^
Other	53.2^a^	53.2^a, b^	54.2^a, b^	55.1^a^
1998–2000 deployment to Bosnia, Kosovo, or Southwest Asia experience^‡^				
No	53.4^a^	53.5^a^	54.5^a^	55.4^a^
Yes	53.6^a^	53.6^a^	54.4^a^	54.9^b^
Length of service, years				
0–3	52.4^a^	53.8^a^	54.8^a^	55.5^a^
4–8	53.5^b^	53.9^a^	54.1^b^	55.1^a^
9–15	54.1^c^	53.9^a^	54.3^a, b^	55.2^a^
≥ 16	54.0^b, c^	52.6^b^	54.6^a^	54.9^a^
Military rank				
Enlisted	52.5^a^	52.4^a^	53.4^a^	54.5^a^
Warrant officer	53.9^b^	53.5^b^	55.3^b^	55.1^a, b^
Commissioned officer	54.2^b^	54.7^c^	54.6^b^	55.9^b^
Branch of service				
Army	53.0^a^	52.5^a^	54.0^a^	54.2^a^
Air Force	54.0^b^	54.2^b^	55.3^b^	55.6^b^
Navy and Coast Guard	53.3^a, c^	54.0^b^	54.6^c^	55.5^b^
Marines	53.8^b, c^	53.5^c^	53.9^a, c^	55.3^b^
Occupational category				
Combat specialists	53.7^a^	53.6^a^	54.6^a^	55.3^a^
Other occupations	53.4^b^	53.5^a^	54.3^b^	55.0^b^

* Medical Outcomes Study 36-Item Short Form Health Survey for Veterans. 1998 general US population means used to calculate mental and physical component summary scores (MCS, PCS)

^† ^Only men with MCS and PCS scores and complete demographic data are reported in this table.

^a, b, c ^Letters that are different indicate statistically significant differences (p < 0.05) of adjusted and weighted means. Same letters indicate no statistically significant differences in means. Tukey's method was used to adjust for multiple comparisons.

## Discussion

Lower health-related quality of life measures have been associated with increased health care utilization [20-22], posttraumatic stress disorder [23], disability [22], behavioral risk factors [22], and mortality [20,22]. This report highlights relatively good health in a large military cohort. Additionally, we have identified a number of sociodemographic and military characteristics that were independently associated with physical and mental health status in service members on active duty and in the US National Guard and Reserves. These included sex, age, rank, educational attainment, marital status, race/ethnicity, duration of military service, component of service, branch of service, and combat occupation specialties. Interestingly, those having recent deployment experience to Southwest Asia, Kosovo, or Bosnia were independently associated with slightly more favorable mental or physical health status as measured by the Medical Outcomes Study Short Form 36-item Survey for Veterans.

Results from this study may be compared to published US general population norms. The PCS and MCS normative US scores have a mean of 50 and a standard deviation of 10, allowing comparison between populations [34]. To interpret differences in mean scores, a difference of five points in the scores is considered clinically and socially meaningful [44]. Age-comparable unadjusted MCS and PCS scores for the Millennium Cohort were higher in comparison with data for the 1998 US general population norms for most age categories [34]. The mean PCS scores for males and females aged 18–34 years for the general population are nearly identical to the mean PCS scores for Millennium Cohort males and females aged 17–24 and 25–34 years of age. However, as age increases the Millennium Cohort mean PCS scores get proportionately higher compared to those of the US population. For example, PCS scores of those aged over 44 years in the Millennium Cohort are about 2 and 4 points higher in men and women, respectively, compared to those aged 44 to 54 years in the US general population. Mean MCS scores are higher in the Millennium Cohort at all age-comparable groups compared to the general US general population. Similar to the PCS scores, the largest differences are seen in the oldest age groups. The youngest Millennium Cohort age groups (17–24 years) have mean MCS scores that are about 2 points higher than the general US population while the scores for the oldest groups (older than 44 years) are 3 points higher in the women and more than 5 points higher in the men. These higher scores, especially in the older age groups, may be due to healthier people entering and remaining in the US military. There are certain physical and mental criteria that must be met to continue service in the US military, which may explain the higher scores when compared to the general US population. Higher MCS and PCS scores in military populations in comparison with US norms have been replicated in other studies where select US military populations were compared with US normative scores. However, as noted above, the statistical significance found comparing Cohort participants to normative values may not indicate clinically significant differences in health status [8,9,45].

Mean PCS and MCS score trends among Millennium Cohort members were similar to those observed in civilian populations. As reported by other researchers [44,46-48], we observed increasing mean MCS scores and decreasing mean PCS scores with increasing age. In this cohort, women reported lower mental and physical functioning than men, similar to their civilian counterparts. A 2002 cross-sectional survey of 4,506 Swedes found that SF-36 scores differed by gender. The authors hypothesize that these differences were due, in part, to gender disparities in work, income, daily living, social life, and expectations between men and women [49]. The authors of this study also noted that there were gender differences in the prevalence and severity of self-reported pain associated with headaches and musculoskeletal disorders, which has also been observed by others for rheumatoid arthritis [50], irritable bowel syndrome [51], fibromyalgia [52], and chronic fatigue syndrome [53,54]. Women serving in the military have reported that they suffer from psychosocial and interpersonal stress associated with being female in the military and that this generally had a stronger impact on women's than on men's mental health [55,56]. This may be supported by the notable lower adjusted mean MCS scores among active-duty women with no high school diploma when compared to their Reserve/National Guard counterparts. We observed similar results to those reported by Voelker et al., who studied 1991 Gulf War-era military personnel [8]. Common findings between the current study and Voelker et al. include decreased mean PCS scores for those who were married or in the Army, and decreased mean MCS scores for service members who were in the Army, divorced, and had shorter lengths of service. In this study, we report increased mean PCS scores with increasing education, which has also been observed in a study of health-related quality of life among a cohort of 1991 Gulf War and Germany-deployed veterans [9].

The Millennium Cohort unadjusted means of the mental and physical component summary scores were much higher than that of Department of Veterans Affairs (VA) populations presenting for care [21,57,58]. The mean MCS and PCS scores for VA enrollees who filled out a questionnaire in 1999 or 2000 were 42.8 and 40.7 for women aged 18 to 44 years and 43.4 and 40.2 for men aged 18 to 44. The MCS scores were about 6 to 10 points higher, while the PCS scores were 11 to 14 points higher among similar Millennium Cohort groups of men and women [58]. This difference is likely due to dissimilarities in VA eligibility criteria that emphasize service-related injury and illness and unmet health service need [21,57]. As the Millennium Cohort ages and members separate or retire from military service and begin to use the VA health care system, comparisons of baseline functional health of these members will enhance the growing knowledge of predictors of mental and physical impairment after military service.

There are notable limitations to these analyses that should be discussed. The Millennium Cohort Study baseline enrollment ended with 36% of those invited consenting to participate in the 21-year study. As with any survey study, response bias and generalizability is a concern and should be investigated when possible. Although participants self-selected in accepting the invitation to become part of the cohort reports of Millennium Cohort baseline data suggest a representative sample of military personnel measured by demographic and health characteristics and reliable health, vaccination, and deployment reporting [16,25-31]. Due to the Cohort being constructed to sample more women, those with recent deployment experience to Southwest Asia, Bosnia, or Kosovo, and Reserve/National Guard, there are compositional differences between the target population and those in military service in October 2000 [16]. However, as demonstrated by the slight difference in weighted means and nonweighted means, these proportional differences have minimal impact when generalizing to the US military. Investigation of a health bias for enrolling in the Millennium Cohort suggested little health differences in responders and nonresponders with respect to hospitalization and outpatient encounters in the year prior to enrollment (data not yet published). Further, reporting bias may have been introduced to these functional health estimates based on an investigation that reported military personnel enrolling soon after the tragic events of September 11, 2001, reported significantly better mental and physical health during the first few months after the attacks than in months prior to the attacks [59]. The finding that those having past deployment experience to Southwest Asia, Kosovo, or Bosnia had slightly more favorable mental or physical health status may simply be due to a selection process where more healthy individuals are deployed. Lastly, although the SF-36 and Veterans SF-36 have undergone reliability investigations and are thought to be reasonable instruments for measuring health perception [33,38,60,61], the use of standardized instruments and self-reported data as a surrogate for clinical health assessment is imperfect.

Despite limitations, our study has a number of strengths. This report documents a very large, population-based investigation of health of current US military members as measured by the Medical Outcomes Study Short Form 36-item Survey for Veterans. The large study population with many demographic characteristics allowed for robust estimates of the two summary scores while adjusting for differences in populations using ANOVA techniques. In addition, the use of standardized instruments allows for the comparison with other populations, such as the US population in general [62] or other military populations [57,63]. Most importantly, the future strength of these data will be in the longitudinal comparison with baseline health during and after deployment as well as in comparison with civilian and other veteran organizations.

## Conclusion

Recent reports have suggested significant mental health morbidity in US military personnel returning from military deployment to Iraq and Afghanistan [64,65], as well as increased risk of neuropsychological compromise after deployment in a cohort of Army personnel [66]. These reports have added to the mounting concern over the physical and mental health of returning deployed personnel as well as the effect this may have on family members, health care utilization, and diminished military readiness for future deployments. In this report, we described the baseline functional health of a large US military cohort as measured by the Medical Outcomes Study Short Form 36-item Survey for Veterans. Our findings suggest, on average, a mentally and physically healthier population than other comparison populations, and will be instrumental in prospectively evaluating health after deployment in a large, population-based military cohort.

## Abbreviations

ANOVA, analysis of variance; MCS, mental component summary score; PCS, physical component summary score; VA, Department of Veterans Affairs

## Competing interests

The author(s) declare that they have no competing interests.

## Authors' contributions

TS, BS, and CL performed the statistical analysis. All authors helped conceive the study, participated in its design and coordination, and helped to draft the manuscript. All authors read and approved the final manuscript.

## Pre-publication history

The pre-publication history for this paper can be accessed here:



## References

[B1] Fukuda K, Nisenbaum R, Stewart G, Thompson WW, Robin L, Washko RM, Noah DL, Barrett DH, Randall B, Herwaldt BL, Mawle AC, Reeves WC (1998). Chronic multisymptom illness affecting Air Force veterans of the Gulf War. JAMA.

[B2] The Iowa Persian Gulf Study Group (1997). Self-reported illness and health status among Persian Gulf War veterans: a population-based study. JAMA.

[B3] Gray GC, Reed RJ, Kaiser KS, Smith TC, Gastanaga VM (2002). The Seabee Health Study: self-reported multi-symptom conditions are common and strongly associated among Gulf War veterans. Am J Epidemiol.

[B4] Kang HK, Mahan CM, Lee KY, Magee CA, Murphy FM (2000). Illnesses among United States veterans of the Gulf War: a population-based survey of 30,000 veterans. J Occup Environ Med.

[B5] Unwin C, Blatchley N, Coker W, Ferry S, Hotopf M, Hull L, Ismail K, Palmer I, David A, Wessely S (1999). The health of United Kingdom servicemen who served in the Persian Gulf War. Lancet.

[B6] Steele L (2000). Prevalence and patterns of Gulf War illness in Kansas veterans: association of symptoms with characteristics of person, place, and time of military service. Am J Epidemiol.

[B7] Unwin C, Blatchley N, Coker W, Ferry S, Hotopf M, Hull L, Ismail K, Palmer I, David A, Wessely S (1999). Health of UK servicemen who served in Persian Gulf War.. Lancet.

[B8] Voelker MD, Saag KG, Schwartz DA, Chrischilles E, Clarke WR, Woolson RF, Doebbeling BN (2002). Health-related quality of life in Gulf War era military personnel. Am J Epidemiol.

[B9] Proctor SP, Harley R, Wolfe J, Heeren T, White RF (2001). Health-related quality of life in Persian Gulf War veterans. Mil Med.

[B10] Hyams KC, Wignall FS, Roswell R (1996). War syndromes and their evaluation: from the U.S. Civil War to the Persian Gulf War. Ann Intern Med.

[B11] Horn O, Hull L, Jones M, Murphy D, Browne T, Fear NT, Hotopf M, Rona RJ, Wessely S (2006). Is there an Iraq war syndrome? Comparison of the health of UK service personnel after the Gulf and Iraq wars. Lancet.

[B12] Hotopf M, Hull L, Fear NT, Browne T, Horn O, Iversen A, Jones M, Murphy D, Bland D, Earnshaw M, Greenberg N, Hughes JH, Tate AR, Dandeker C, Rona R, Wessely S (2006). The health of UK military personnel who deployed to the 2003 Iraq war: a cohort study. Lancet.

[B13] Haley RW (1998). Point: bias from the "healthy-warrior effect" and unequal follow-up in three government studies of health effects of the Gulf War [see comments]. Am J Epidemiol.

[B14] Smith B, Smith TC, Ryan MA, Gray GC (2006). A comparison of the postdeployment hospitalization experience of U.S. military personnel following service in the 1991 Gulf War, Southwest Asia after the Gulf War, and Bosnia. J Occup Environ Hyg.

[B15] Gray GC, Knoke JD, Berg SW, Wignall FS, Barrett-Connor E (1998). Counterpoint: Responding to suppositions and misunderstandings. Am J Epidemiol.

[B16] Ryan MA, Smith TC, Smith B, Amoroso P, Boyko EJ, Gray GC, Gackstetter GD, Riddle JR, Wells TS, Gumbs G, Corbeil TE, Hooper TI (2007). Millennium Cohort: enrollment begins a 21-year contribution to understanding the impact of military service. J Clin Epidemiol.

[B17] Secretary of Defense (1998). Report to the Committee on National Security, House of Representatives, and the Armed Services Committee, U.S. Senate, on effectiveness of medical research initiates regarding Gulf War illnesses.

[B18] Committee on Measuring the Health of Gulf War Veterans IM, Hernandez LM, Durch JS, Blazer II DG, Hoverman IV (1999). Gulf War veterans: measuring health..

[B19] Kazis LE, Ren XS, Lee A, Skinner K, Rogers W, Clark J, Miller DR (1999). Health status in VA patients: results from the Veterans Health Study. Am J Med Qual.

[B20] Singh JA, Nelson DB, Fink HA, Nichol KL (2005). Health-related quality of life predicts future health care utilization and mortality in veterans with self-reported physician-diagnosed arthritis: the Veterans Arthritis Quality of Life Study. Semin Arthritis Rheum.

[B21] Singh JA, Borowsky SJ, Nugent S, Murdoch M, Zhao Y, Nelson DB, Petzel R, Nichol KL (2005). Health-related quality of life, functional impairment, and healthcare utilization by veterans: Veterans' Quality of Life Study. J Am Geriatr Soc.

[B22] Centers for Disease Control and Prevention (2000). Measuring Healthy Days.

[B23] Butterfield MI, Forneris CA, Feldman ME, Beckham JC (2000). Hostility and functional health status in women veterans with and without posttraumatic stress disorder: a preliminary study. J Trauma Stress.

[B24] Dillman DA (1978). Mail and telephone surveys: the total design method.

[B25] LeardMann CA, Smith B, Smith TC, Wells TS, Ryan MA (2007). Smallpox vaccination: Comparison of self-reported and electronic vaccine records in the Millennium Cohort Study. Hum Vaccin.

[B26] Riddle JR, Smith TC, Smith B, Corbeil TE, Engel CC, Wells TS, Hoge CW, Adkins J, Zamorski M, Blazer D (2007). Millennium Cohort: The 2001-2003 baseline prevalence of mental disorders in the U.S. military. J Clin Epidemiol.

[B27] Smith B, Leard CA, Smith TC, Reed RJ, Ryan MA (2007). Anthrax vaccination in the Millennium Cohort; validation and measures of health. Am J Prev Med.

[B28] Smith B, Smith TC, Gray GC, Ryan MA (2007). When Epidemiology Meets the Internet: Web-based Surveys in the Millennium Cohort Study. Am J Epidemiol.

[B29] Smith TC, Jacobson IG, Smith B, Hooper TI, Ryan MA, Team FT (2007). The occupational role of women in military service: validation of occupation and prevalence of exposures in the Millennium Cohort Study. Int J Environ Health Res.

[B30] Smith TC, Smith B, Jacobson IG, Corbeil TE, Ryan MA (2007). Reliability of Standard Health Assessment Instruments in a Large, Population-Based Cohort Study. Ann Epidemiol.

[B31] Smith B, Wingard DL, Ryan MAK, Macera CA, Patterson TL, Slymen DJ, for the Millennium Cohort Study Team US military deployment during 2001-2006: comparison of subjective and objective data sources in a large prospective health study.. Ann Epidemiol.

[B32] Office of the Under Secretary of Defense Personnel and Readiness (2001). DoD Instruction 1312.1-I, DoD Occupational Conversion Index, 03/2001.

[B33] Ware JE, Sherbourne CD (1992). The MOS 36-Item Short-Form Health Survey (SF-36). I. conceptual framework and item selection. Med Care.

[B34] Ware JE, Kosinski M (2001). SF-36 Physical & Mental Health Summary Scales: A Manual for Users of Version 1..

[B35] Ware JE, Kosinski M, Keller SD (1994). SF-36 physical and mental health summary scales: a user's manual.

[B36] Perlin J, Kazis LE, Skinner K, Ren XS, Lee A, Rogers WH, Spiro A, Selim A, Miller D (2000). Health status and outcomes of veterans: physical and mental component summary scores, Veterans SF-36, 1999 Large Health Survey of Veteran Enrollees. Executive report.

[B37] Kazis LE, Miller DR, Clark J, Skinner K, Lee A, Rogers W, Spiro A, Payne S, Fincke G, Selim A, Linzer M (1998). Health-related quality of life in patients served by the Department of Veterans Affairs: results from the Veterans Health Study. Arch Intern Med.

[B38] Kazis LE, Miller DR, Clark JA, Skinner KM, Lee A, Ren XS, Spiro A, Rogers WH, Ware JE (2004). Improving the response choices on the veterans SF-36 health survey role functioning scales: results from the Veterans Health Study. J Ambul Care Manage.

[B39] Kazis LE, Lee A, Spiro A, Rogers W, Ren XS, Miller DR, Selim A, Hamed A, Haffer SC (2004). Measurement comparisons of the Medical Outcomes Study and Veterans SF-36 Health Survey. Health Care Financ Rev.

[B40] Tukey JW (1994). The Collected Works of John W. Tukey VIII. Multiple Comparisons. 1948, 1983.

[B41] Kramer CY (1956). Extension of multiple range tests to group means with unequal numbers of replications. Biometrics.

[B42] Woodruff RF (1971). A simple method for approximating the variance of a complicated estimate. J Am Stat Assoc.

[B43] SAS Institute Inc. (2002). SAS/STAT® software version 9.0..

[B44] Hopman WM, Towheed T, Anastassiades T, Tenenhouse A, Poliquin S, Berger C, Joseph L, Brown JP, Murray TM, Adachi JD, Hanley DA, Papadimitropoulos E (2000). Canadian normative data for the SF-36 health survey. Canadian Multicentre Osteoporosis Study Research Group. CMAJ.

[B45] Eisen SA, Kang HK, Murphy FM, Blanchard MS, Reda DJ, Henderson WG, Toomey R, Jackson LW, Alpern R, Parks BJ, Klimas N, Hall C, Pak HS, Hunter J, Karlinsky J, Battistone MJ, Lyons MJ (2005). Gulf War veterans' health: medical evaluation of a U.S. cohort. Ann Intern Med.

[B46] Hopman WM, Berger C, Joseph L, Towheed T, VandenKerkhof E, Anastassiades T, Adachi JD, Ioannidis G, Brown JP, Hanley DA, Papadimitropoulos EA (2006). The natural progression of health-related quality of life: results of a five-year prospective study of SF-36 scores in a normative population. Qual Life Res.

[B47] Hemingway H, Nicholson A, Stafford M, Roberts R, Marmot M (1997). The impact of socioeconomic status on health functioning as assessed by the SF-36 questionnaire: the Whitehall II Study. Am J Public Health.

[B48] Hemingway H, Stafford M, Stansfeld S, Shipley M, Marmot M (1997). Is the SF-36 a valid measure of change in population health? Results from the Whitehall II Study. BMJ.

[B49] Bingefors K, Isacson D (2004). Epidemiology, co-morbidity, and impact on health-related quality of life of self-reported headache and musculoskeletal pain─a gender perspective. Eur J Pain.

[B50] Lapsley HM, March LM, Tribe KL, Cross MJ, Courtenay BG, Brooks PM (2002). Living with rheumatoid arthritis: expenditures, health status, and social impact on patients. Ann Rheum Dis.

[B51] Lee OY, Mayer EA, Schmulson M, Chang L, Naliboff B (2001). Gender-related differences in IBS symptoms. Am J Gastroenterol.

[B52] Yunus MB (2002). Gender differences in fibromyalgia and other related syndromes. J Gend Specif Med.

[B53] Buchwald D, Pearlman T, Kith P, Schmaling K (1994). Gender differences in patients with chronic fatigue syndrome. J Gen Intern Med.

[B54] Cannon JG, St Pierre BA (1997). Gender differences in host defense mechanisms. J Psychiatr Res.

[B55] Bray RM, Fairbank JA, Marsden ME (1999). Stress and substance use among military women and men. Am J Drug Alcohol Abuse.

[B56] Vogt DS, Pless AP, King LA, King DW (2005). Deployment stressors, gender, and mental health outcomes among Gulf War I veterans. J Trauma Stress.

[B57] Kazis LE, Miller DR, Skinner KM, Lee A, Ren XS, Clark JA, Rogers WH, Spiro A, Selim A, Linzer M, Payne SM, Mansell D, Fincke RG (2004). Patient-reported measures of health: The Veterans Health Study. J Ambul Care Manage.

[B58] Frayne SM, Parker VA, Christiansen CL, Loveland S, Seaver MR, Kazis LE, Skinner KM (2006). Health status among 28,000 women veterans. The VA Women's Health Program Evaluation Project. J Gen Intern Med.

[B59] Smith TC, Smith B, Corbeil TE, Riddle JR, Ryan MA, for the Millennium Cohort Study Team (2004). Self-reported mental health among US military personnel prior and subsequent to the terrorist attacks of September 11, 2001. J Occup Environ Med.

[B60] Brazier JE, Harper R, Jones NM, O'Cathain A, Thomas KJ, Usherwood T, Westlake L (1992). Validating the SF-36 health survey questionnaire: new outcome measure for primary care. BMJ.

[B61] Jenkinson C, Wright L, Coulter A (1994). Criterion validity and reliability of the SF-36 in a population sample. Qual Life Res.

[B62] Ware JE, Kosinski M, Gandek B (2000). SF-36 Health Survey: manual and interpretation guide..

[B63] Zamorski MA Evaluation of an enhanced post-deployment health screening program for Canadian forces members deployed on Operation APOLLO (Afghanistan/SW Asia). http://www.forces.gc.ca/health/information/op_health/op_apollo/engraph/op_apollo_toc_e.asp.

[B64] Hoge CW, Castro CA, Messer SC, McGurk D, Cotting DI, Koffman RL (2004). Combat duty in Iraq and Afghanistan, mental health problems, and barriers to care.. N Engl J Med.

[B65] Hoge CW, Auchterlonie JL, Milliken CS (2006). Mental health problems, use of mental health services, and attrition from military service after returning from deployment to Iraq or Afghanistan. JAMA.

[B66] Vasterling JJ, Proctor SP, Amoroso P, Kane R, Heeren T, White RF (2006). Neuropsychological outcomes of Army personnel following deployment to the Iraq war. JAMA.

